# Cysteine Conjugation: An Approach to Obtain Polymers with Enhanced Muco- and Tissue Adhesion

**DOI:** 10.3390/ijms252212177

**Published:** 2024-11-13

**Authors:** Marta Chrószcz-Porębska, Agnieszka Gadomska-Gajadhur

**Affiliations:** Faculty of Chemistry, Warsaw University of Technology, Noakowskiego 3 Street, 00-664 Warsaw, Poland; marta.porebska@pw.edu.pl

**Keywords:** L-cysteine, polymer-L-cysteine conjugates, amino acid conjugation, mucoadhesion, tissue adhesion, polymer modification

## Abstract

The modification of polymers towards increasing their biocompatibility gathers the attention of scientists worldwide. Several strategies are used in this field, among which chemical post-polymerization modification has recently been the most explored. Particular attention revolves around polymer-L-cysteine (Cys) conjugates. Cys, a natural amino acid, contains reactive thiol, amine, and carboxyl moieties, allowing hydrogen bond formation and improved tissue adhesion when conjugated to polymers. Conjugation of Cys and its derivatives to polymers has been examined mostly for hyaluronic acid, chitosan, alginate, polyesters, polyurethanes, poly(ethylene glycol), poly(acrylic acid), polycarbophil, and carboxymethyl cellulose. It was shown that the conjugation of Cys and its derivatives to polymers significantly increased their tissue adhesion, particularly mucoadhesion, stability at physiological pH, drug encapsulation efficiency, drug release, and drug permeation. Conjugates were also non-toxic toward various cell lines. These properties make Cys conjugation a promising strategy for advancing polymer applications in drug delivery systems and tissue engineering. This review aims to provide an overview of these features and to present the conjugation of Cys and its derivatives as a modern and promising approach for enhancing polymer tissue adhesion and its application in the medical field.

## 1. Introduction

Polymers have been used as biomaterials since the 1940s [1]. Since then, their applications have expanded significantly to include a wide range of synthetic and natural polymers [2,3,4,5]. They found applications in various medical fields, including drug delivery systems for controlled and targeted drug release [6,7], scaffolds and biodegradable matrices for tissue regeneration [8,9,10], orthopedic and dental implants [11,12], medical devices such as catheters and stents [13,14], and wound care [15,16].

Polymeric materials are essential to modern medicine due to their customizable physicochemical and mechanical properties. Depending on the application, they can be engineered to be flexible or rigid, hydrophobic or hydrophilic, and degradable or non-degradable [17]. Compared to conventional ceramic and metallic biomaterials, polymers are lighter, more affordable to produce, easier to fabricate, and thermal, making them ideal for sterilization [18].

Despite these advantages, polymers often lack sufficient biocompatibility [19], which is crucial for materials intended for use in the human body. Non-biocompatible compounds can trigger inflammatory responses, exert cytotoxic effects that lead to tissue necrosis, and exhibit low compatibility with biological tissues. Modern polymeric biomaterials research focuses on improving biocompatibility through various strategies [20]. One approach involves blending synthetic polymers with natural biocompatible materials such as collagen, chitosan, or hyaluronic acid or combining polymers with biocompatible ceramics or metals [21,22,23]. Another strategy involves surface modification and functionalization through chemical and physical processes, such as attaching bioactive molecules or incorporating anti-inflammatory agents [24,25,26,27,28].

**Figure 1:**
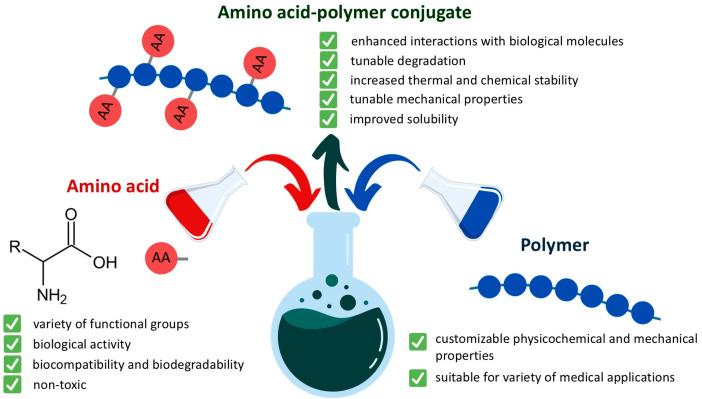
Graphical representation of polymer–amino acid conjugates.

Particular attention in this field revolves around polymer–amino acid and polymer–peptide conjugates [29,30,31,32,33,34]. Polymer–amino acid conjugates are hybrid materials formed by covalent or non-covalent interactions between polymers and biomolecules [32]. These hybrid materials combine polymers’ desirable properties with amino acids’ biological functionality [35,36] (Figure 1).

Amino acids are the building blocks of proteins. They play vital roles in numerous biological processes within living organisms, including protein synthesis and enzyme production [37,38,39]. They also contribute to metabolic pathways [40,41] and immune system health [42,43]. Since amino acids are naturally present in the body, they are non-toxic, biocompatible, and biodegradable [44]. Conjugating amino acids with polymers provides various benefits, such as introducing functional groups that enhance interactions with biological molecules [45,46], improving drug delivery [36,47], modulating polymer degradation [48], increasing thermal and chemical stability and improving the solubility of hydrophobic polymers [34,36,49]. These features make polymer–amino acid conjugates valuable in medical applications, including drug delivery systems [50,51], scaffolds [52], and hydrogels [53].

**Figure 2:**
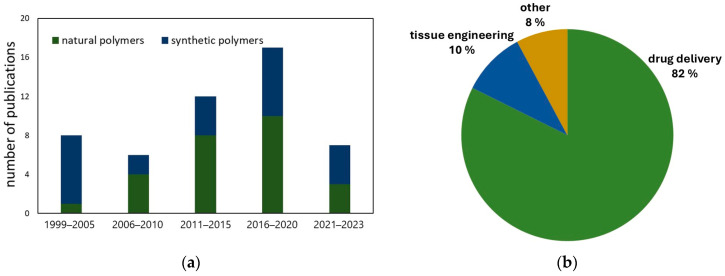
(**a**) The relation between the number of publications regarding modifying polymers with L-cysteine or its derivatives and the publication year. The graph includes the division into natural and synthetic polymers. (**b**) The application of L-cysteine–polymer conjugation in the medical field.

L-cysteine (Cys) is a particularly noteworthy amino acid for polymer modification. Like other amino acids, it contains an alpha carbon bonded to a hydrogen atom (H), a carboxyl group (-COOH), and an amine group (-NH_2_) [37,54]. Cys is the only natural amino acid with a unique side chain with a thiol group (-CH_2_SH), giving it distinct properties [55] (Figure 2). The thiol group is highly reactive, with strong nucleophilic characteristics [56], and can form disulfide bonds with other Cys molecules, stabilizing the three-dimensional structure of proteins, antibodies, and enzymes [57]. Cys also plays a role in maintaining cellular redox balance, contributes to the synthesis of glutathione, and binds metal ions, which is crucial for enzymatic reactions involving metal ion cofactors [58,59,60].

The reactivity and variety of functional groups in the Cys molecule, particularly its thiol group, make it ideal for polymer modification. Polymer–Cys conjugation has become increasingly important in two primary fields: medicine and metal treatment. In the medical field, the thiol group plays a crucial role in improving the tissue adhesion of polymers [50,51,61,62]. This is especially valuable in applications such as tissue engineering and drug delivery systems, where strong adhesion to biological tissues is critical for the material’s functionality. Polymer–Cys conjugates also gain attraction in metal treatment, mainly due to Cys’ high affinity to metal ions [56,60]. The thiol group readily binds to metals, which makes them highly effective in water treatment for removing heavy metals [63,64,65,66].

The unique properties of Cys and its derivatives, including their ability to enhance tissue adhesion, have encouraged scientists to explore their potential in the field of polymers for medical applications. This field has evolved significantly over the past 20 years, with natural and synthetic polymers undergoing conjugations (Figure 2a). Conjugates have primarily been tested for their potential use in drug delivery systems. Additionally, several studies have presented their use as scaffolds for tissue engineering and wound healing (Figure 2b).

**Figure 3:**
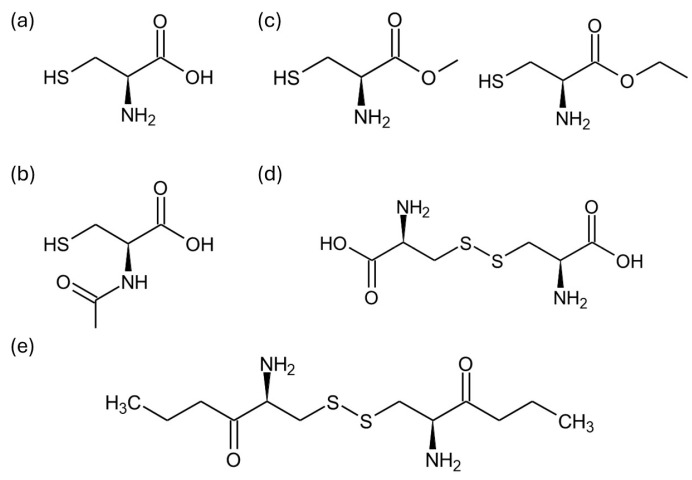
Chemical structure of L-cysteine and its derivatives used for polymer modification: (**a**) L-cysteine, (**b**) N-acetyl-L-cysteine, (**c**) L-cysteine methyl and ethyl esters, (**d**) L-cystine, and (**e**) L-cystine diethyl ester.

Both unmodified Cys and its derivatives, such as N-acetyl-L-cysteine, L-cysteine esters, L-cystine, and L-cystine esters (Figure 3), have been explored in various contexts.

Despite the increasing interest in Cys-modified polymers, much of the existing literature has concentrated on the broader class of polymer–peptide conjugates, often overlooking the role of Cys in polymer modification. This review addresses this gap by comprehensively summarizing the research on polymer–Cys conjugates. It aims to highlight the employed synthesis techniques, the functional properties introduced by Cys, and the potential benefits these materials offer, particularly in medical applications. By summarizing the recent advancements in polymer–Cys conjugation, this review presents the conjugation of Cys and its derivatives as a modern and promising approach for enhancing polymer tissue adhesion and its application in the medical field.

## 2. Role of L-Cysteine in Tissue Adhesion

Tissue adhesion is the process by which natural or synthetic material attaches to a biological surface [67]. It plays a critical role in various biomedical applications, including drug delivery and tissue engineering [68,69,70]. In particular, when adhesion involves a mucosal membrane, the process is called mucoadhesion, a subset of tissue adhesion that enables more targeted applications in mucosal drug delivery systems [71].

**Figure 4:**
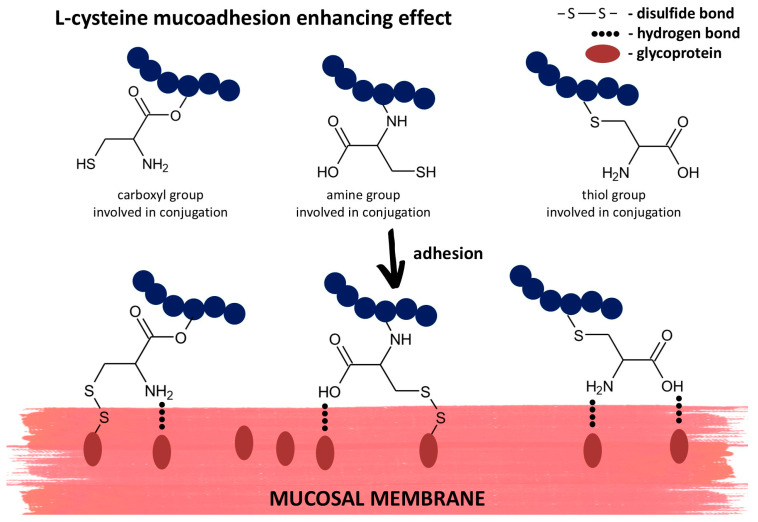
Graphical representation of L-cysteine role in tissue adhesion on the example of mucoadhesion.

Conjugation of L-cysteine to polymers has shown promise for enhancing tissue adhesion due to the variety of its functional groups, which can facilitate covalent and non-covalent interactions with biological tissues (Figure 4) [72].

L-cysteine contains thiol groups that can readily form disulfide bonds with other thiol-containing molecules, particularly those found in tissue proteins such as collagen, elastin, and fibronectin, as well as with the mucin glycoproteins in mucus. These disulfide bonds, which are covalent in nature, are often formed with the cysteine-rich subdomains in proteins’ extracellular matrix. As disulfide bonds are stable under physiological conditions, they offer durable adhesion, making them ideal for applications that require prolonged tissue attachment. In addition to covalent bonding, L-cysteine’s amino and carboxyl groups can form hydrogen bonds with various functional groups in tissue proteins. Although they are weaker than the covalent bonds, they still contribute significantly to overall adhesion by promoting molecular contact and enhancing the stability of the attachment. Hydrogen bonds help create cohesive interaction between the adhesive and tissues, especially when other binding mechanisms are impossible. Finally, the swelling behavior of L-cysteine–polymer conjugates plays a pivotal role in enhancing tissue adhesion. Cysteine-based materials tend to swell upon exposure to body fluids, allowing the material to spread evenly across the tissue surface. This improves the likelihood of disulfide bonding and other interactions as more thiol groups become available to bond with the tissue.

## 3. L-Cysteine Conjugation Approaches

Amino acids can be conjugated to polymers through covalent or non-covalent interactions [32].

Non-covalent conjugation relies on reversible interactions such as hydrogen bonding, electrostatic forces, hydrophobic interaction, and van der Waals forces. These interactions are typically reversible and can be enhanced by pretreating the polymer surface. The main advantages of non-covalent conjugation are its simplicity and reversibility. However, the resulting conjugates generally are characterized by low stability.

On the contrary, more common covalent conjugation involves the formation of stable, permanent covalent bonds between the amino acid and polymer [73]. There are three primary strategies for covalent conjugation: *grafting to*, *grafting from*, and *grafting through* [29,32].

In the *grafting to* strategy, the polymer is synthesized through a specific polymerization process, and the amino acid is then grafted onto the polymer backbone or chain ends via reactions between the functional groups of the polymer and the amino acid. Due to the variety of available functional groups in both amino acid and polymers, it can be achieved by thiol-ene, thiol-yne, Michael addition, native chemical ligation, amidation, pyridyl disulfide, epoxy ring opening, huisgen dipolar cycloaddition, thio-bromo click ligation, reductive amination, and oxime ligation reaction [32]. This approach is also known as post-polymerization modification and is the most widely used method for amino acid–polymer conjugation.

The *grafting from* strategy incorporates the amino acid moiety directly into the polymerization initiator molecule. This strategy involves common polymerization techniques such as Atom Transfer Radical Polymerization (ATRP), Nitroxide-Mediated Radical Polymerization (NMP), and reversible Addition Fragmentation Chain-Transfer (RAFT) [32].

The latter strategy, *grafting through*, involves the polymerization in which amino acid moiety is a part of the macromonomer. It employs methods like RAFT, ATRP, Ring Opening Polymerization (ROP), Ring Opening Metathesis Polymerization (ROMP), Nitroxide Mediated Polymerization (NMP), and simple polycondensation [32].

**Figure 5:**
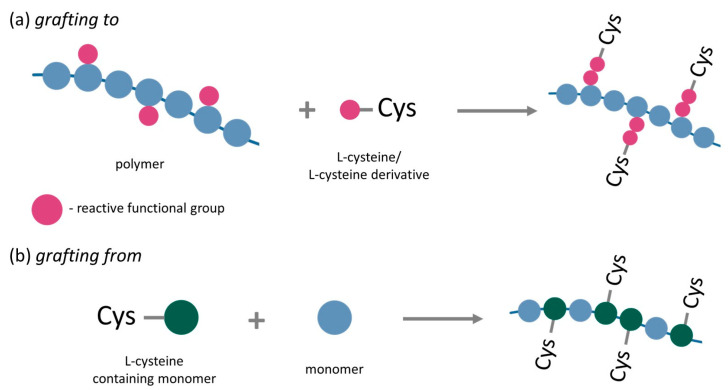
Strategies for conjugating L-cysteine and its derivatives to polymers: (**a**) *grafting to* (post-polymerization) and (**b**) *grafting from*.

For conjugating L-cysteine (Cys) and its derivative to polymers, the *grafting to* strategy (Figure 5) has been the most used. This is primarily due to the diverse functional groups in Cys—thiol (-SH), amine (-NH_2_), and carboxylic (-COOH). The reactivity of those functional groups varied depending on the reaction type and conditions, which allowed for tailoring the properties of the resulting amino acid–polymer conjugate.

Cys and its derivatives were conjugated to natural polymers, mainly polysaccharides, via post-polymerization modification (Table 1). It was achieved through an amidation reaction between the amine group of Cys/Cys derivative and the carboxylic group of polysaccharides [68,69,70,71,72,73,74,75,76,77,78,79,80,81,82,83,84,85,86,87,88,89,90,91,92,93,94,95,96,97,98,99]. The reaction often requires the additional step of carboxylic group preactivation with the coupling agents. It was needed to increase its reactivity towards the amino acid amine group, and therefore, as shown in the study of Kafedjiiski et al. [85], to increase the reaction efficiency. As a coupling agent, a system based on (3-dimethylaminopropyl)carbodiimide hydrochloride/N-hydroxy succinimide (EDAC/NHS) was commonly used [74,75,76,77,78,79,80,81,82,83,84,85,86,88,90,95,96,97,98,99]. The resulting conjugates, called thiomers, featured free thiol groups prone to instability under physical pH and oxidative stress conditions. Thus, they were often protected with mercaptonicotinic acid compounds [75,81,84,88].

An alternative method for conjugating Cys to polysaccharides, proposed by Zhang et al. [99], involves conjugating Cys to hyaluronic acid through ether linkage between the hyaluronic acid hydroxyl group and an epoxy group of Cys-based compound, leaving a free amine and thiol group that can be further functionalized.

In the context of synthetic polymers, various reactions have been employed to conjugate Cys. They were mainly obtained via the *grafting to* strategy [51,98,101,102,103,104,105,106,107,108,109,110,111,112,113,114,115,116,117], although some studies also explored the *grafting through* strategy [118,119,120,121]. Common reactions used for grafting Cys on polymers include thiol-ene [102,103,104,105], amidation [51,98,101,106,107,108,111,112,113,114,115,116,117], imidation [109], and thio-Michael addition [110] (Table 2).

The thiol group of Cys was conjugated to an unsaturated polyester double bond, both for native Cys [105] and its N-acetyl derivative (NAC) [102,103,104]. These reactions were either initiated thermally or through photoinitiation using AIBN [102,103,104] or DMPA [105]. Polyesters were also modified via thio-Michael addition with L-cysteine methyl ester (CysOMe) [110]. Unlike the thiol-ene reaction, thio-Michael addition allowed for polyester conjugation without an initiator and at low temperatures.

More commonly, the amine group of the Cys moiety was used to conjugate synthetic polyesters. Cys was conjugated to polyesters [106], carboxymethyl cellulose (CMC) [98,111,117], poly(acrylic acid) (PAA) [51,101,112,113,114,116], and polycarbophil (PCP) [101,111,115]. The conjugation of Cys to CMC, PAA, and PCP involved the reaction of the Cys amine group with the carboxylic group of the polymer and, like with natural polymers, required preactivation of the carboxylic moiety with EDAC [51,98,101,111,112,113,114,115,116,117]. For polyester conjugation, a stable amide bond was formed between the Cys amine and polymer ester groups [106]. By amide bond formation, NAC was also conjugated to poly(ethylene glycol) (PEG) [108]. In this case, the hydroxyl group of PEG stearate was transformed to be more reactive towards amidation, the p-nitrophenyl group. The imine [109] and ester [107] bonds were used for Cys conjugation less frequently. By amination of the surface of PU with 3-aminopropyltriethoxysilane with subsequent Schiff’s base formation with glutaraldehyde, it was possible further to conjugate Cys via imine bond formation [108]. On the other hand, a simple esterification reaction between the NAC carboxylic group and PEG stearate hydroxyl moiety [107] was employed.

The grafting trough strategy has been utilized to conjugate L-cystine (CC) and L-cystine ester to PU [119,120] and polyesters [118] as well as to conjugate S-tert butylmercapto L-cysteine (CysStBu) to PEG [114]. In this approach, CC [119], L-cystine diethyl ester (CCOEt) [120] was reacted with prepolymers, specifically 1,6-hexamethylene diisocyanate (HMDI)/poly(ε-caprolactam) (PCL) [119,120] or HMDI/PEG [119], obtained via the addition reaction. By applying melt polycondensation, CC was conjugated to polyesters [118]. This two-step process involves synthesizing the cystine monomer with dicarboxylic esters and urethane functional groups, followed by melt polycondensation with diols. In another example, anionic ROP was used to create a PEG and polycysteine (PCys) block copolymer [121] by polymerizing CysStBu N-carboxyanhydride with an amino-terminated PEG macroinitiator [121].

## 4. Cysteine–Polymer Conjugate

### 4.1. Natural Polymers

Natural polymers are essential components of the biological systems from which they are extracted [122] and have garnered significant attention due to their inherent biodegradability and biocompatibility [3,123]. These attributes make them highly suitable for various medical applications, including developing hydrogels, drug delivery systems, wound healing materials, and tissue engineering scaffolds for skin and bone repair, as well as in cell encapsulation and biofabrication [122]. Natural polymers commonly utilize polysaccharides, such as hyaluronic acid, chitosan, and alginate, alongside proteins like collagen, gelatin, silk, and fibrin [3,124].

Polysaccharides, particularly hyaluronic acid, chitosan, and alginate were repeatedly subjected to thiolation with L-cysteine (Cys) and its derivatives. The primary goal of thiolation was not to enhance the biocompatibility of these polymers but to improve their stability under physiological pH conditions and to enhance their mucoadhesive properties (Table 3).

Thiolated polymers, often referred to as thiomers, exhibit distinctive mucoadhesive capabilities due to the presence of free thiol groups. These groups can form intra- and interchain disulfide bonds within the polymer network and with cysteine residues in the mucus glycoprotein [62,125]. This contributes to the increased stability of the polymer–mucus interaction, extending the residence time of drug delivery systems on mucosal surfaces. This prolonged residence time offers a significant advantage in drug delivery applications, as it enhances drug absorption through the mucus layer, thereby improving the therapeutic efficacy of the encapsulated drug [125].

#### 4.1.1. Hyaluronic Acid

Hyaluronic acid (HA) is recognized for its remarkable properties, including high biocompatibility, viscosity, elasticity, low immunogenicity, and exceptional water retention capacity. In the human body, HA plays an important structural and hydration role [77], maintaining hydrated and stable extracellular spaces and acting as a lubricant in joints [126]. However, HA is sensitive to harsh conditions, including strong acids, bases, heat, and free radicals, and is prone to enzymatic degradation [120,127]. It limits its application in systems with prolonged release time [76]. Additionally, neat HA lacks specific tissue adhesion sites, restricting its use in the medical field [128]. To overcome these limitations, numerous strategies have been explored [126,129,130], among which particular attention has been given to thiolation [131].

Cys and its derivatives have been widely used for the thiolation of HA. Numerous studies have investigated the thiolation of HA using Cys [76,78,82,84,100] and L-cysteine ethyl ester (CysOEt) [65,74,77,79,80,81,83,85]. In many cases, CysOEt was chosen over Cys due to its greater reactivity and physiological pH. Since thiolate anion is a reactive form of thiomers, the amino acid should ionize at physiological pH (6.7 to 7.4 [74]). Concerning this, CysOEt was chosen as it has a lower pKa value (pKa of 7.4) than neat Cys (pKa of 8) [75].

The first attempts to thiolate HA with CysOEt were reported by Kafedjiiski et al. [85]. Since then, a notable increase in publications regarding this field has occurred, driven by the work of Laffleur and colleagues [74,75,79].

Performed studies showed that HA thiolated with CysOEt (HA-CysOEt) did not exhibit cytotoxic effects on colon carcinoma cells [74,75,77,79,80,81]. However, it was observed that the preactivation of the thiol group with 6-mercaptonicotinamide could increase cytotoxicity due to the aromatic groups in its structure [81]. Covalent attachment of CysOEt improved water sorption [74,75,79,81] and stability of HA under physiological pH [74,75,81]. Thiolated HA showed enhanced resistance to enzymatic degradation, particularly from enzymes like lysozyme, amylase, and hyaluronidase [77], due to the formation of inter- and intra-disulfide bonds [74]. Additionally, thiolated HA showed superior mucoadhesive properties. It revealed increased adhesion to the nasal [83], buccal [74,79], intestinal [77], and vaginal [81] mucosa, as well as to simulated saliva fluid [75]. Importantly, thiol-protected HA-CysOEt showed higher mucoadhesion than its unprotected counterpart [81]. The enhanced stability of HA-CysOEt contributed to improved drug release profiles, with better control observed for drugs like rotigonite [74] and sulforhodamine [79]. Conjugation with CysOEt also enhanced the permeation of drugs like rotigonite [74], curcumin [79], sulforhodamine [80], and fluorescein isothiocyanate-dextran [80] through mucus.

While the thiol group is known to be unstable in physiological pH and oxidative conditions [76], limiting its application in liquid formulations, studies by Laffleur et al. [83] and Pereira de Sousa et al. [84] demonstrated that protecting the thiol groups in HA-CysOEt can maintain stability for up to four weeks at pH of 7.4 and under oxidative conditions [84]. Moreover, protected HA-CysOEt also exhibited enhanced mucoadhesion [83,84] without increasing cytotoxicity [84].

Although most studies used CysOEt to thiolate HA, modification with Cys was also investigated [76]. HA-Cys conjugates showed enhanced mucoadhesion [76,78], greater resistance to enzymatic degradation by hyaluronidase [76], trypsin and chymotrypsin [78], better-controlled drug release [76,78], and higher water uptake [76]. Similar to the HA-CysOEt conjugates, HA-Cys did not exhibit cytotoxicity [78]. Moreover, studies highlighted the influence of the molecular weight of thiolated HA on its properties, including stability, water uptake, degradation, and drug release, suggesting that molecular weight can be used to tailor Ha-Cys-based drug delivery systems [76,82]. HA-Cys conjugates have also been evaluated for potential use as injectable, in situ-forming hydrogels [100]. An innovative method for conjugating Cys without involving its amine and thiol groups has shown promise in producing materials suitable for further modification. It was proved that HA-Cys conjugates can be cross-linked via native chemical ligation and Michale addition, forming hydrogels with improved stability and drug delivery capabilities.

The chemical structures of HA modified with Cys and its derivatives can be found in the Supplementary Materials (Figure S1).

#### 4.1.2. Chitosan

Chitosan (CS) is known for its high antibacterial activity, biocompatibility, biodegradability, and non-toxicity [132]. It is applicable in many areas of medicine, including drug delivery, gene therapy, immunity, and tissue engineering [133,134,135]. Among these, drug delivery is the most prominent. Despite its outstanding biological properties, there are certain limitations to using CS in drug delivery. First, CS showed limited solubility. CS is only soluble at acidic pH, which reduces its drug loading capacity and excludes its uses in a physiological pH as it precipitates [136]. Second, CS exhibits mucoadhesion only in acidic environments, limiting its applicability in intranasal drug delivery, where the pH typically ranges between 5.5 and 6.5.

Thiolation of CS with Cys and its derivatives has emerged as an effective solution to these issues [61]. Various studies have explored the modification of CS with Cys [88,89,90,92,93] and its N-acylated derivative (NAC) [87,90,91,92,93,94,99]. These thiolated CS conjugates have been validated to be used in a wide range of medical applications, including drug delivery systems [87,88,89,90,91,93,99], scaffolds [86], and homeostatic dressings [92].

In drug delivery, thiolated CS has been extensively studied for use in ophthalmic [91], oral [87,90], and intranasal [89] applications. The thiolated derivatives of CS have been particularly investigated as coatings for lipid-based formulations. For instance, Liu et al. demonstrated that CS-NAC, used as a coating for lipid nanocarriers with encapsulated curcumin, slowed the release of curcumin and enhanced its transcorneal penetration [91]. Similarly, Du et al. explored CS-NAC and CS-Cys as coatings for hybrid nanohydrogels based on NaOH-modified casein [93] or β-lactoglobulin [90], which effectively entrapped both hydrophobic and hydrophilic compounds and provided controlled drug release. Nanaki et al. studied the use of CS-Cys in the intranasal administration of galantamine nanoparticles [89] combined with carbon materials such as dots or hierarchical porous carbons. These CS-Cys-based materials exhibited higher drug-loading capacities and slower drug release. CS-Cys with a 6-mercaptonicotinic acid/Cys-protected thiol group were also used as a coating for solid lipid nanoparticles [88]. It turned out that this process increases mucus interaction and mucus diffusion. It was also observed by Schmitz et al. that the CS-NAC conjugate could enhance rotigonite and fluorescein isothiocyanate-dextran permeation through rat intestines [87].

In addition to drug delivery, thiolated CS has been utilized in the development of homeostatic dressings [92]. Meena et al. produced semi-interpenetrating cryogels with reduced cytotoxicity. The enhanced water solubility of CS-Cys allowed for the exclusion of acidic solvents from cryogel preparation, using water instead, thereby reducing toxicity.

Thiolated CS has also shown promise in tissue engineering, particularly for cartilage tissue repair. Fernanda et al. developed CS-Cys-based hydrogel scaffolds, which exhibited lower swelling and higher degradation stability than scaffolds made from native CS. Moreover, these scaffolds were highly porous and compatible with tissues [87].

Chemical structures of CS modified with Cys and its derivatives can be found in the Supplementary Materials (Figure S2).

#### 4.1.3. Alginate

Alginate (Alg) is characterized by biocompatibility, low toxicity, hydrophilicity, gel-forming ability, and porosity [137,138,139]. Despite these advantages, Alg lacks adhesive properties in cells and tissues [140], which limits its effectiveness in specific biomedical applications. To address this limitation, efforts have been made to modify Alg with Cys [95,96,97,98].

Alginate–Cys (Alg-Cys) conjugates have demonstrated significant improvements in tissue adhesion properties. Compared to native Alg, these conjugates exhibit enhanced mucoadhesion [96,97], water uptake [95,97], and stability [97]. This improvement is attributed to the rapid formation of disulfide bonds between the thiol group of Alg-Cys and mucus, which helps protect the thiol group from oxidation [97]. Additionally, Alg-Cys conjugates have proven effective in forming drug-loaded nanoparticles with higher drug encapsulation efficiency, as seen in the sulfamethoxazole/trimethoprim system [96]. Moreover, the conjugation of Cys to Alg enhanced drug permeation, which was confirmed in vitro [95] and in vivo [98], leading to increased release of atorvastatin calcium [95] and sulforhodamine [98].

The chemical structure of Alg modified with Cys can be found in the Supplementary Materials (Figure S3).

### 4.2. Synthetic Polymers

Synthetic polymers, unlike their natural counterparts, do not occur in nature but are produced through chemical reactions using substances derived from petroleum oil. These polymers offer superior mechanical characteristics and enhanced thermal stability compared to natural polymers [21], often similar to tissues. Therefore, synthetic polymers have found widespread use in various medical applications, including tissue engineering [141], wound healing [142,143], drug delivery systems [144,145], and implants [12,146]. One of the critical advantages of synthetic polymers is their versatility [147]. They can be engineered to possess a wide range of properties, making them adaptable to specific applications. Their physicochemical and mechanical properties can be tailored by adjusting their synthesis conditions, chemical structure, or molecular weight. However, a significant limitation of synthetic polymers is that they lack cell adhesion sites, which hinders their interactions with tissues [147].

To address this issue, synthetic polymers have been chemically modified by conjugation with L-cysteine (Cys) and its derivatives (Table 4). These modifications introduce functional groups into the polymer structure, enhancing their adhesion to biological tissues. Among the synthetic polymers frequently subjected to such modifications were polyesters, polyurethanes, and poly(acrylic acid), along with its derivatives, all of which improved performance in medical applications.

#### 4.2.1. Polyesters

The group of polyesters encompasses a wide range of biodegradable polymers with tunable physical and mechanical properties [149], making them highly suitable for various biomedical applications [150,151]. Recent studies on polyester modification focused on enhancing their tissue adhesion [149,152] and developing polyesters as anticancer drug carriers [153].

A key modification involves the thiol-ene reaction between the functional groups of polyesters and the thiol group of Cys moieties. For instance, poly(globalide-co-ε-caprolactone) copolyester modified with Cys (PGlCl-Cys) [105] and N-acetyl-L-cysteine (NAC) (PGlCl-NAC) [103,104] demonstrated increased hydrophilicity and reduced crystallinity compared to unmodified polyester. Scaffolds based on PGlCl-NAC blended with poly(ε-caprolactone) (PCL) were found to be non-toxic to fibroblasts and promoted high cell viability and proliferation [103]. Additionally, the NAC molecule introduced antioxidant properties to the polyesters, which are particularly important for tissue engineering [104]. PGlCl-Cys, when used as a coating for superparamagnetic iron oxide nanoparticles, exhibited enhanced biomolecule affinity, facilitating the conjugation of folic acid and methotrexate [105]. The drug-loaded nanoparticles demonstrated controlled release, indicating potential effectiveness in cancer treatment. In further studies, NAC was used as a drug itself. Mrówka et al. modified aliphatic polysuccinates with allyl pendant groups [102]. Modified polyester showed improved solubility and no cytotoxicity to normal human keratinocytes while showing anticancer activity against squamous and melanoma carcinoma cells. Polyesters conjugated with Cys were also explored as drug delivery systems at low pH. Microparticles of poly(L-lactide)-b-poly(2-(methacryloyloxy)ethyl phosphorylcholine)-st-(methacrylic acid NHS ester), encapsulating rhodamine B and modified with Cys, provided controlled drug release over 89 days, with accelerated release at acidic pH [106]. In another approach, unsaturated polyesters of maleic acid containing L-cysteine methyl ester (CysOMe) moieties exhibited thermal degradability [110]. At the temperature corresponding to human organisms, polyester showed low degradation (after 4 h, less than 20% of the polymer underwent degradation).

Anantharaj et al. [116] employed a different strategy to implement the amino acid moiety in polyesters. By *grafting through*, cationic Cys-based polyesters were obtained. Polyesters demonstrated no cytotoxicity to breast cancer cell lines and degraded into low molecular weight compounds that could be easily removed from the body.

The chemical structures of polyesters modified with Cys and its derivatives can be found in the Supplementary Materials (Figure S4).

#### 4.2.2. Polyurethanes

Polyurethanes are inherent to modern medicine. They are composed of hard and soft segments, depending on which their properties can be tailored [154]. Polyurethanes are notable for their superior mechanical strength compared to other synthetic polymers used in medicine. Its combination with high biocompatibility and biodegradability makes polyurethanes suitable for various medical applications [154,155,156].

Studies on polyurethane–Cys conjugates aimed to increase their hemocompatibility [109] and degradability [119,120]. One of the primary challenges in using polyurethanes for blood-contacting devices is platelet adhesion. This issue can be mitigated by modifying polyurethanes to release nitric oxide (NO), as NO is known to inhibit platelet aggregation. Simple thiols, such as Cys, are particularly useful for this purpose due to their ability to transfer NO from S-nitroproteins and release it from their structures. This was confirmed by Duan et al. [109], who demonstrated that polyurethane, provided by DuPont company, modified with Cys, reduced platelet adhesion by 50%. Polyurethanes, conjugated with Cys derivative, showed improved degradation. The well-known mechanisms of polyurethane degradation are hydrolysis, oxidation, and light degradation, occurring over an extended period. In the studies by Lu et al. [119] and Wang et al. [120], the introduction of cystine dimethyl ester (CCOEt) as a chain extender to the polyurethane based on 1,6-hexamethylene diisocyanate (HMDI) as a hard segment, and poly(ε-caprolactam) (PCL) as a soft segment, makes them sensitive to reductive degradation. As shown, modified polyurethane underwent degradation induced by glutathione, with a rapid molecular weight decrease within the first days. Moreover, modified polyurethanes were non-toxic to human umbilical vein endothelial cells [119,120], promoted cell proliferation [120], and showed favorable mechanical properties [120].

The chemical structures of polyurethanes modified with Cys derivatives can be found in the Supplementary Materials (Figure S5).

#### 4.2.3. Poly(ethylene glycol)

Poly(ethylene glycol) (PEG) is the most common synthetic polymer used in drug delivery systems [157,158]. The reasons behind this are its unique properties, such as solubility in water and low cytotoxicity. PEG increases the water solubility of hydrophobic drugs, provides greater physical and thermal stability of drug formulations, and prevents their aggregation. Moreover, PEG’s solubility in various organic solvents facilitates the modification of its functional group [157,159].

Studies on PEG conjugation with N-acetyl-L-cysteine (NAC) (NAC-PEG) were focused on its application as coatings for nanostructured lipid carriers (NLCs) to enhance drug delivery in oral [107] and ocular [108] applications. In these studies, NAC-PEG conjugates were used as NLC surface modifiers. Modified NLC showed high curcumin [107] and cyclosporine [108] encapsulation efficiency and sustained drug release profiles. Furthermore, modified NLC showed enhanced permeation through mucus [107] and improved mucoadhesion [107,108] while exhibiting no irritant effect on rabbit eyes [108].

Apart from NLC modifiers, PEG-modified with Cys has been developed as an antioxidant [121]. A block copolymer of PEG and polycysteine (PCys) with a protected thiol group exhibited the ability to form stable nanomicelles in an aqueous environment due to its amphiphilic character. These nanomicelles maintained stability across a pH range from 2 to 8 and demonstrated low toxicity to mouse colon carcinoma and normal human umbilical vein endothelial cell lines. Notably, they also exhibited an antitumor effect. This shows that despite the redox conditions exerted by the tumor cells, Cys-based conjugates could be effective in cancer treatment.

The chemical structures of poly(ethylene glycol) modified with Cys and its derivatives can be found in the Supplementary Materials (Figure S6).

#### 4.2.4. Poly(acrylic acid) and Polycarbophil

Poly(acrylic acid) (PAA) and its cross-linked counterpart, polycarbophil (PCP), are widely recognized for their ability to form hydrogels, biocompatibility, and biodegradability [160]. These polymers exhibit mucoadhesive properties due to the carboxylic groups in their structure, which interact with mucus via hydrogen bonding [161]. However, their mucoadhesion is based on relatively weak hydrogen interactions. This prompted researchers to enhance it by conjugating L-cysteine (Cys) to their structure [51,112,113,114,116,148].

The conjugation of Cys to PCP (PCP-Cys) significantly improved its stability [148], water uptake [111], and mucoadhesion [101,111,148], particularly at an acidic pH of 5 [111]. PCP-Cys conjugates also showed enhanced drug permeation across the intestinal mucosa, as observed with sodium fluorescein and peptide drugs like bacitracin and isoline [115]. Similar to PCP-Cys, PAA-Cys conjugates showed increased stability [115,116,148], water uptake [115,116,148], drug permeation [111], and adhesion to mucus [114,116,148], with mucoadhesion levels surpassing those of PCP-Cys [148]. It was further observed that the mucoadhesion of PAA-Cys was related to the thiolation degree [113,114] and pH [116]. This is not surprising, as mucoadhesion depends on the disulfide bond formation between thiol groups of conjugate and mucus and the reactive form of thiomers is an anion that is favorably formed at lower pH. However, high degrees of thiolation did not necessarily improve drug penetration, probably due to increased self-cross-linking and viscosity, which limit polymer interpenetration [113]. Moreover, studies revealed a correlation between PAA’s molecular weight and its conjugates’ properties [148]. Higher molecular weights enhanced stability, water uptake, and mucoadhesion [148]. To further optimize PAA-Cys, Iqbal et al. [51] protected the thiol groups in 2-mercaptonicotinic acid, resulting in conjugates with improved stability, higher water uptake, and mucoadhesion. They were also non-toxic to Caco-2 cells.

Although previously mentioned works focused primarily on drug delivery applications of PCP in PAA, recent work has extended its use into tissue engineering. Bolanta et al. [112] developed printable hydrogels based on PAA-Cys conjugates for application as scaffolds in cell culture. Hydrogels, obtained by adding acrylic acid to PAA-Cys and subsequent cross-linking, demonstrated stability for up to two weeks, high water uptake, and non-toxic effects on human retinal pigment epithelial cells, showing their potential for use in tissue engineering.

The chemical structures of PAA and PCP modified with Cys and its derivatives can be found in the Supplementary Materials (Figure S7).

#### 4.2.5. Carboxymethylcellulose

Carboxymethyl cellulose (CMC), a semi-synthetic natural polymer [162], has found extensive applications in drug delivery systems, tissue engineering, and wound dressings [162,163,164].

Recent studies by Laffleur et al. explored the potential of CMC conjugated with Cys (CMC-Cys) to enhance its mucoadhesive properties [98,111,117]. The CMC-Cys conjugate demonstrated several advantages, including increased water uptake, improved stability, and superior adhesion to buccal mucosa [111,117]. Notably, the conjugate exhibited no cytotoxic effect on Caco-2 cells [117] and facilitated enhanced sulforhodamine permeation [98,117], underscoring its potential for mucosal drug delivery.

The chemical structure of CMC modified with Cys can be found in the Supplementary Materials (Figure S8).

## 5. Outcomes and Future Prospectives

The need to improve tissue adhesion of polymers for medical applications is under investigation by scientists worldwide. Several strategies are employed to achieve this goal; among them, the conjugation of amino acids is gathering increasing attention. L-cysteine (Cys) has gained attention because of the presence of amine (-NH_2_), carboxylic (-COOH), and thiol (-SH) groups, which together provide a multifaceted mechanism for improving polymer–tissue interactions.

The articles cited in this review showed that Cys–polymer conjugates can be applied, especially in tissue engineering and drug delivery, the latter being underlined in the vast majority.

Conjugation of Cys and its derivatives to both natural and synthetic polymers showed that Cys–polymer conjugates: (i) allow for obtaining drug delivery systems with prolonged residence time, (ii) have superior control over drug release profiles, (iii) exhibited increased water uptake attributed to improved hydrophilicity and swelling behavior, (iv) are non-toxic, (v) have improved drug loading capacity, (vi) show mucoadhesion even in a broad range of pH, and (vii) allow for tailoring their properties like stability, water uptake, degradation rates by modulating their molecular weight. All those improvements were thanks to the functional groups in the Cys molecule. Due to the thiol group, Cys and its derivatives can form strong covalent bonds with Cys-rich subdomains in tissues and mucus glycoproteins. In contrast, the amine and carboxylic groups facilitate hydrogen bond formation with tissue. The combination of covalent disulfide bonds with non-covalent hydrogen bonding, intensified by the swelling of Cys–polymer conjugates in an aqueous environment, contributes to a significant increase in general tissue adhesion, making such conjugates particularly attractive for drug delivery systems. Conjugating Cys or its derivatives to polymers enabled overcoming problems associated with modern drug delivery. Due to the high stability of disulfide bonds, Cys–polymer conjugates can be applied in drug delivery systems for acidic environments such as the gastrointestinal tract and to deliver therapeutic peptides and proteins. Apart from ocular, vaginal, intranasal, and gastrointestinal drug delivery, Cys–polymer conjugates can act as a drug itself, as Cys and its derivatives are known for their therapeutic activity.

Cys–polymer conjugates also contribute to the field of tissue engineering applications. By conjugating Cys or its derivative, it was possible to obtain scaffolds: (i) with unique pore distribution, (ii) non-toxic to various human cell lines, (iii) having lower crystallinity and higher hydrophilicity, (iv) enhancing cell proliferation, and (v) possessing tunable mechanical properties. These properties offer significant advantages for tissue engineering applications. Hydrophilic surfaces promote strong tissue adhesion and enhance integration with surrounding tissues. The unique pore distribution within the material provides sites for cells to attach, encouraging cell proliferation across the scaffold. Additionally, adjusting mechanical properties allows the scaffold’s strength and flexibility to be tailored to suit specific application sites.

While beneficial in biomedical applications, Cys–polymer conjugates have limitations, which could be an interesting issue for further studies.

The stability of Cys–polymer conjugates should be more extensively investigated. Although conjugates with appropriate stability in physiological conditions were achieved, it was possible due to the protection of the Cys thiol group. This was achieved by introducing mercaptonicotinic acid or its derivatives. It was shown that despite higher stability, protected conjugates showed an increase in cytotoxicity. In this context, further studies should be undertaken in which mercaptonicotinic acid will be replaced by less harmful compounds. Extensive studies should also be undertaken on the potential for toxic byproducts. During degradation, cysteine-modified polymers may release byproducts, including thiol-containing compounds. In some cases, these by-products could have adverse biological effects, possibly resulting in cytotoxicity, especially if they accumulate in sensitive tissues. While cysteine–polymer conjugates have shown promise in targeting tumor cells, their degradation can be unpredictable depending on the body’s redox condition. Tumor cells showed elevated levels of reactive oxygen species (ROP) compared to normal cells. High levels of ROS disrupt redox balance, causing oxidative stress conditions, which may promote the degradation of cysteine–polymer conjugates. While tumors exhibit high ROS concentration, they also showed an increased concentration of glutathione (GSH). Cys–polymer conjugates can be designed to respond to GSH for controlled degradation. However, increased GSH concentration can significantly promote their degradation and thus reduce the antitumor activity. Although the potential of Cys–polymer conjugates in cancer treatment was investigated, the problem of its degradation under disturbed redox conditions was barely studied.

## Figures and Tables

**Table 1:** Synthesis of L-cysteine–natural polymer conjugates.

Polymer	Modifier	Strategy	Reaction Type	Refs.
hyaluronic acid	L-cysteine	*grafting to*	amidation with the carboxylic group preactivation	[76,78,82]
			amidation with thiol group protection	[84]
			ether bond formation	[100]
	L-cysteine ethyl ester	*grafting to*	amidation with the carboxylic group preactivation	[74,77,79,80,83,85]
			amidation with thiol group protection	[75,81]
			amidation	[89]
chitosan	L-cysteine	*grafting to*	amidation with the carboxylic group preactivation	[86,90,92,93,99]
			amidation with thiol group protection	[88]
	N-acetyl-L-cysteine	*grafting to*	amidation with the carboxylic group preactivation	[87,90,91,94]
alginate	L-cysteine	*grafting to*	amidation with the carboxylic group preactivation	[95,96,97,98]

**Table 2:** Synthesis of L-cysteine–synthetic polymer conjugates.

Polymer		Modifier	Strategy	Reaction Type	Refs.
polyester	poly(globalide-co-ε-caprolactone)	L-cysteine	*grafting to*	photo initiated thiol-ene reaction	[105]
		N-acetyl-L-cysteine	*grafting to*	thermally initiated thiol-ene reaction	[103,104]
	polysuccinates	N-acetyl-L-cysteine	*grafting to*	thermally initiated thiol-ene reaction	[102]
	poly(L-lactide)-b-poly(2-(methacryloyloxy)ethyl phosphorylcholine)-st-(methacrylic acid NHS ester)	L-cysteine	*grafting to*	amidation	[106]
	di(10-undecenyl) maleate	L-cysteine methyl ester	*grafting to*	thiol-Michael addition	[110]
	cystine monomer	L-cystine	*grafting from*	melt polycondensation	[118]
polyurethane	by DuPont company	L-cysteine	*grafting to*	imidation	[109]
	HMDI/PCL	L-cystine	*grafting from*	polyaddition	[119]
		L-cystine diethyl ester	*grafting from*	polyaddition	[120]
	HMDI/PEG	L-cystine	*grafting from*	polyaddition	[119]
poly(ethylene glycol)		L-cysteine	*grafting to*	addition with thiol group preactivation	[107]
			*grafting from*	anionic ROP	[121]
		N-acetyl-L-cysteine	*grafting to*	addition with thiol group preactivation	[108]
poly(acrylic acid)		L-cysteine	*grafting to*	addition with thiol group preactivation	[51,101,112,113,114,116]
polycarbophil		L-cysteine	*grafting to*	addition with thiol group preactivation	[101,111,115]
carboxymethyl cellulose		L-cysteine	*grafting to*	addition with thiol group preactivation	[98,101,111,117]

**Table 3:** Application and properties of conjugates based on natural polymers.

Conjugate	Application	Properties ^a^	Refs.
hyaluronic acid/L-cysteine	drug delivery	Property—HA molecular weight dependency; 2-fold higher enzymatic degradation stability; 11-fold higher swelling ratio; 2-fold prolonged ovalbumin drug release.	[76]
		4.82-fold ^b^ increased mucoadhesion with small intestine rat segment; Non-toxic to Madin–Darby canine kidney (MDCK) cell line.	[78]
	not defined	Property—HA molecular weight dependency; 3-fold higher swelling ratio.	[82]
hyaluronic acid/L-cysteine with 2-mercaptonicotinic acid protected thiol group	drug delivery	52-fold ^c^ higher mucoadhesion with mucus; 2.2-fold ^d^ and 1.8-fold ^d^ higher mucoadhesion with small intestine and palpebral conjunctiva; Non-toxic to colorectal adenocarcinoma (Caco-2) cell line; Stability at pH 7.4 and under oxidative stress.	[84]
hyaluronic acid/L-cysteine ethyl ester	drug delivery	12.16-fold ^c^ and 2.82-fold ^d^ higher mucoadhesion with buccal mucosa; 1.49-fold higher stability; 3.47-fold improved swelling ratio; 1.29-fold more controlled rotigonite release; 1.18-fold higher rotigonite permeation through buccal mucosa; Non-toxic to Caco-2 cell line.	[74]
		2.17-fold ^c^ higher mucoadhesion with mucus; 11.5-fold higher stability; Non-toxic to Caco-2 cell line.	[77]
hyaluronic acid/L-cysteine ethyl ester	drug delivery	3.4-fold ^d^ higher mucoadhesion with buccal mucosa; 2.75-fold higher swelling capacity; 1.6-fold more controlled curcumin (CUR) release; 4.4-fold higher CUR permeation through buccal mucosa; Non-toxic to Caco-2 cell line.	[81]
		1.28-fold and 1.47-fold in vitro permeation enhancement for sulforhodamine (SR 101) and fluorescein isothiocyanate-dextran (FD4) through Caco-2 cells; 1.9-fold and 1.32-fold enhancement permeation of SR and rhodamine123 (Rh123) ex vivo; Non-toxic to Caco-2 cell line.	[82]
		3.26-fold ^c^ and 1.4-fold ^e^ mucoadhesive with nasal mucosa; 7.6-fold higher stability; 1.75-fold longer residence time on the nasal mucosa.	[83]
		6.5-fold ^c^ higher mucoadhesion with intestinal porcine mucosa; 1.5-fold higher stability; Sustained FD4 drug release.	[85]
hyaluronic acid/L-cysteine ethyl ester with a 6-mercaptonicotinamide-protected thiol group	drug delivery	18-fold ^d^ higher mucoadhesion with simulated saliva fluid; 24.5-fold higher swelling ratio; 24-fold higher stability; Non-toxic to Caco-2 cell line.	[75]
		4-fold ^c^ and 1.6-fold ^d^ higher mucoadhesion with bovine vaginal mucosa; 3.6-fold higher stability; Non-toxic to Caco-2 cell line.	[81]
chitosan/L-cysteine	Tissue engineering	0.87-fold lower swelling ratio; 2-fold higher gel fraction; Uniform pore size distribution—pore size of 223 μm; Non-toxic to human embryonic kidney 293 (HEK 293) and human osteosarcoma (HOS) cell lines.	[86]
	drug delivery	Controlled and sustained release of egg white-derived peptide (EWDP) and CUR; 50–81% and 41–57% entrapment efficiency of EWDP and CUR; 4.56-fold enhanced permeation of pentapeptide isolated from EWDP through Caco-2 cell line.	[90]
		Controlled and sustained release of EWDP and CUR; Stability in acidic pH; 45–58% and 48–63% entrapment efficiency of EWDP and CUR.	[93]
	homeostatic dressings	2.42-fold lower hydrophobicity index; 1.56-fold higher swelling ratio; 1.20-fold higher compressive modulus; Non-toxic to mouse fibroblast (3T3-L1) cells.	[92]
chitosan/L-cysteine with 6-mercaptonicotinic acid/L-cysteine protected thiol group	drug delivery	3-fold ^c^ higher mucoadhesion with mucus.	[88]
chitosan/N-acetyl-L-cysteine	drug delivery	Enhanced Rh123 and FD4 permeation through rat intestine; Non-toxic to Caco-2 cell line.	[87]
		Controlled and sustained release of EWDP and CUR; 51–89% and 42–57% entrapment efficiency of EWDP and CUR; 2.04-fold enhanced permeation of pentapeptide isolated from EWDP through Caco-2 cell line.	[90]
chitosan/N-acetyl-L-cysteine	drug delivery	6-fold longer CUR release; 4.2-fold enhanced CUR permeation through excised corneas.	[91]
		Controlled and sustained release of EWDP and CUR; Stability in acidic pH; 39 to 67% and 51 to 63% entrapment efficiency of EWDP and CUR.	[93]
		6.4-fold enhanced CUR permeation through rabbit cornea; Prolonged and sustained CUR release; No irritation observed for rabbit eye	[94]
		50-fold ^c^ and 8.3-fold ^d^ higher mucoadhesion with mucus.	[99]
alginate/L-cysteine	drug delivery	7.7-fold higher swelling ratio; 5-fold higher atorvastatin calcium release.	[95]
		3-fold ^d^ higher mucoadhesion with gelatin/collagen; 1.1-fold higher sulfamethoxazole/trimethoprim (SMZ/TMP) loading efficiency.	[96]
		1.6-fold ^c^ and 4-fold ^d^ higher mucoadhesion with intestinal mucosa; 3-fold higher stability; 2.5-fold higher swelling ratio.	[97]
		3.0-fold (in vitro) and 1.9-fold (in vivo) enhanced permeation of SR 101 through rat intestinal mucosa.	[98]

^a^ compared to native polymer. ^b^ determined on self-made equipment. ^c^ determined by rheological assay. ^d^ determined by tensile strength assay. ^e^ determined by bioadhesive examination.

**Table 4:** Application and properties of conjugates based on synthetic polymers.

Conjugate	Application	Properties ^a^	Refs.
poly(globalide-co-ε-caprolactone)/ L-cysteine	drug delivery	Lower crystallinity; Lower hydrophobicity; Conjugation efficiency of folic acid (FA) and methotrexate (MTX) above 65%.	[105]
poly(globalide-co-ε-caprolactone)/ N-acetyl-L-cysteine	tissue engineering	Lower crystallinity; Lower hydrophobicity; 2-fold enhanced proliferation of mouse fibroblast (McCoy) cell line; Non-toxic to McCoy cell line.	[103]
	drug delivery tissue engineering	Lower crystallinity; Lower hydrophobicity.	[104]
polysuccinates/N-acetyl-L-cysteine	drug delivery	Improved solubility; Non-toxic to normal human keratinocytes (HaCaT) and normal human fibroblast (NHDF) cell lines; Anticancer activity against squamous carcinoma (SCC-25) and melanoma (Me45) cell lines.	[102]
poly(L-lactide)-b-poly(2-(methacryloyloxy)ethyl phosphorylcholine)-st-(methacrylic acid NHS ester)/L-cysteine	drug delivery	Controlled and gradual release of rhodamine B (RhB) drug over 89 days at an acidic pH of 2 and physiological pH of 7.4.	[106]
di(10-undecenyl) maleate/L-cysteine methyl ester	drug delivery	Sensitive to thermal degradation at a temperature between 25 and 50 °C.	[110]
polyester based on L-cystine monomer and diols	not defined	Degradation by dithiothreitol (DTT) to low molecular weight compounds; Non-toxic to breast cancer (MCF-7) cell line.	[117]
polyurethane by DuPont company/ L-cysteine	blood contacting devices	2-fold lower platelet adhesion; L-cysteine moiety participated in nitric oxide (NO) transportation from S-nitroso albumin, followed by the subsequent release of NO.	[109]
HMDI/PCL/L-cystine methyl ester	tissue engineering drug delivery	Sensitive to enzymatic degradation; Thermal stability up to 180 °C; Non-toxic to human umbilical vein endothelial (HUVEC) cell line.	[119]
HMDI/PCL/L-cystine ethyl ester	not defined	Sensitive to enzymatic degradation; Thermal stability up to 180 °C; Non-toxic to human umbilical vein endothelial (HUVEC) cell line.	[120]
HMDI/PEG/L-cystine methyl ester	tissue engineering drug delivery	Sensitive to enzymatic degradation; Thermal stability up to 220 °C; Non-toxic to human umbilical vein endothelial (HUVEC) cell line.	[119]
poly(ethylene glycol)/N-acetyl-L-cysteine	drug delivery	CUR encapsulation efficiency > 90%; Sustained and prolonged CUR release; 3-fold enhanced CUR permeation through rat intestinal.	[107]
copolymer of poly(ethylene glycol) and polycysteine	drug delivery	Improved stability in the presence of bovine serum albumin at physiological pH; Non-toxic to mouse colon (colon-26) cell line (in vitro) and mice (in vivo).	[121]
poly(ethylene glycol)/L-cysteine	drug delivery	1.6-fold higher mucoadhesion with porcine mucosa; 1.5-fold prolonged precorneal cyclosporine A retention time; Non-irritant to the rabbit eyes.	[108]
poly(acrylic acid)/L-cysteine	drug delivery	452-fold ^b^ and 3-fold ^c^ higher mucoadhesion with intestinal mucosa; 182-fold higher swelling ratio; 69.1-fold higher stability; Non-toxic to Caco-2 cell line.	[51]
		40-fold ^b^ and 4-fold ^c^ higher mucoadhesion with intestinal mucosa; 10-fold higher stability at physiological pH; 1.76-fold and 1.73-fold enhanced sodium fluorescein permeation through Caco-2 and rat small intestine.	[113]
		25-fold ^b^ and 3-fold ^c^ higher mucoadhesion with intestinal mucosa; High swelling ratio.	[114]
poly(acrylic acid)/L-cysteine	drug delivery	7-fold ^b^ and 4-fold ^c^ higher mucoadhesion with intestinal mucosa at acidic pH; 5-fold ^b^ higher mucoadhesion with intestinal mucosa at physiological pH; 4-fold higher swelling ratio; 5-fold higher stability at acidic pH; 20-fold higher stability at physiological pH.	[116]
		1.3-fold ^b^ and 3-fold ^c^ higher mucoadhesion with intestinal mucosa; 20-fold higher swelling ratio; More than 6-fold higher stability in physiological pH.	[148]
	tissue engineering drug delivery	Tunable mechanical properties; Stability for a minimum of 2 weeks; Promoted cell adhesion; Non-toxic to retinal pigment epithelial (RPE1) cell line.	[112]
polycarbophil/L-cysteine	drug delivery	2-fold ^c^ higher mucoadhesion with intestinal mucosa; 2-fold higher swelling ratio.	[101]
		1.4-fold higher swelling ratio at physiological pH; 2.5-fold ^c^ higher mucoadhesion with intestinal mucosa at a pH of 5; 1.25-fold ^c^ higher mucoadhesion with intestinal mucosa at physiological pH.	[111]
		1.6-fold enhanced permeation of sodium fluoresceine through the intestinal mucosa; 1.36-fold enhanced permeation of bacitracin through the intestinal mucosa; 1.33-fold enhanced permeation of insulin through the intestinal mucosa.	[115]
		1.8-fold ^b^ and 3-fold ^c^ higher mucoadhesion with intestinal mucosa; 1.6-fold higher swelling ratio; More than 6-fold higher stability at physiological pH.	[148]
carboxymethyl cellulose/L-cysteine	drug delivery	1.8-fold enhanced SR 101 permeation through rat small intestine.	[98]
		1.6-fold ^c^ higher mucoadhesion with intestinal mucosa at a pH of 5.	[111]
		2.7-fold ^b^ and 1.6-fold ^c^ improved mucoadhesion with buccal mucosa; 2.8-fold higher stability; 2.5-fold higher swelling ratio; Non-toxic to Caco-2 cell line.	[117]

^a^ compared to native polymer. ^b^ determined by rheological assay. ^c^ determined by tensile strength assay.

## Supplementary Materials

The following supporting information can be downloaded at https://www.mdpi.com/article/10.3390/ijms252212177/s1.
