# Supplementary material for: Cysteine Conjugation: An Approach to Obtain Polymers with Enhanced Muco- and Tissue Adhesion
Source: Int J Mol Sci. 2024 Nov 13;25(22):12177. doi: 10.3390/ijms252212177 (PMC11594736; doi:10.3390/ijms252212177)
Supplement: Supplementary file 1 [file ijms-25-12177-s001.zip › ijms-3303692-supplementary.docx]

**Supplementary Materials**

Cysteine Conjugation: An Approach to Obtain Polymers with Enhanced Muco- and Tissue Adhesion

Marta Chrószcz-Porębska and Agnieszka Gadomska-Gajadhur *

Faculty of Chemistry, Warsaw University of Technology, Noakowskiego 3 Street, 00-664 Warsaw, Poland; marta.porebska@pw.edu.pl

***** Correspondence: agnieszka.gajadhur@pw.edu.pl


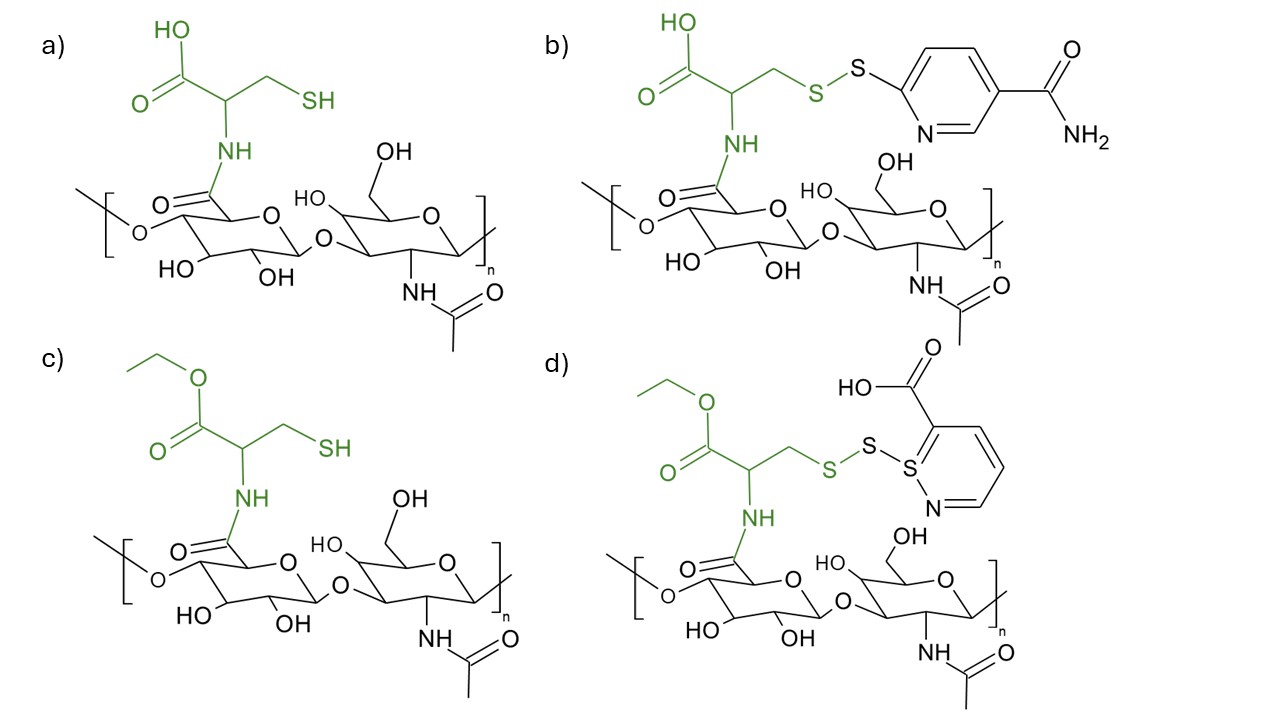


**Figure S1.** Chemical structure of hyaluronic acid-based conjugates: a) hyaluronic acid/L-cysteine, b) hyaluronic acid/L-cysteine with 2-mercaptonicotinic acid protected thiol group, c) hyaluronic acid/L-cysteine ethyl ester, d) hyaluronic acid/L-cysteine ethyl ester with 6-mercaptonicotinamide protected thiol group.


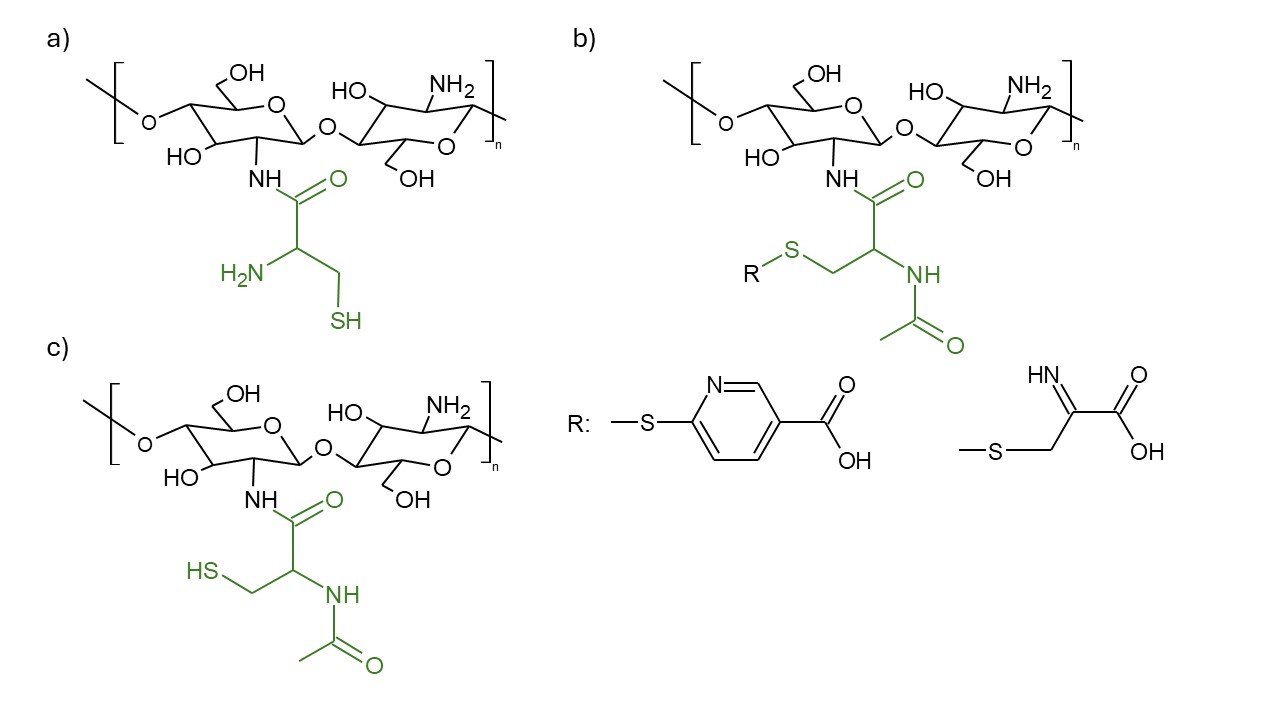


**Figure S2.** Chemical structure of chitosan-based conjugates: a) chitosan/L-cysteine, b) chitosan/L-cysteine with 6-mercaptonicotinic acid protected thiol group, c) chitosan/N-acetyl-L-cysteine.


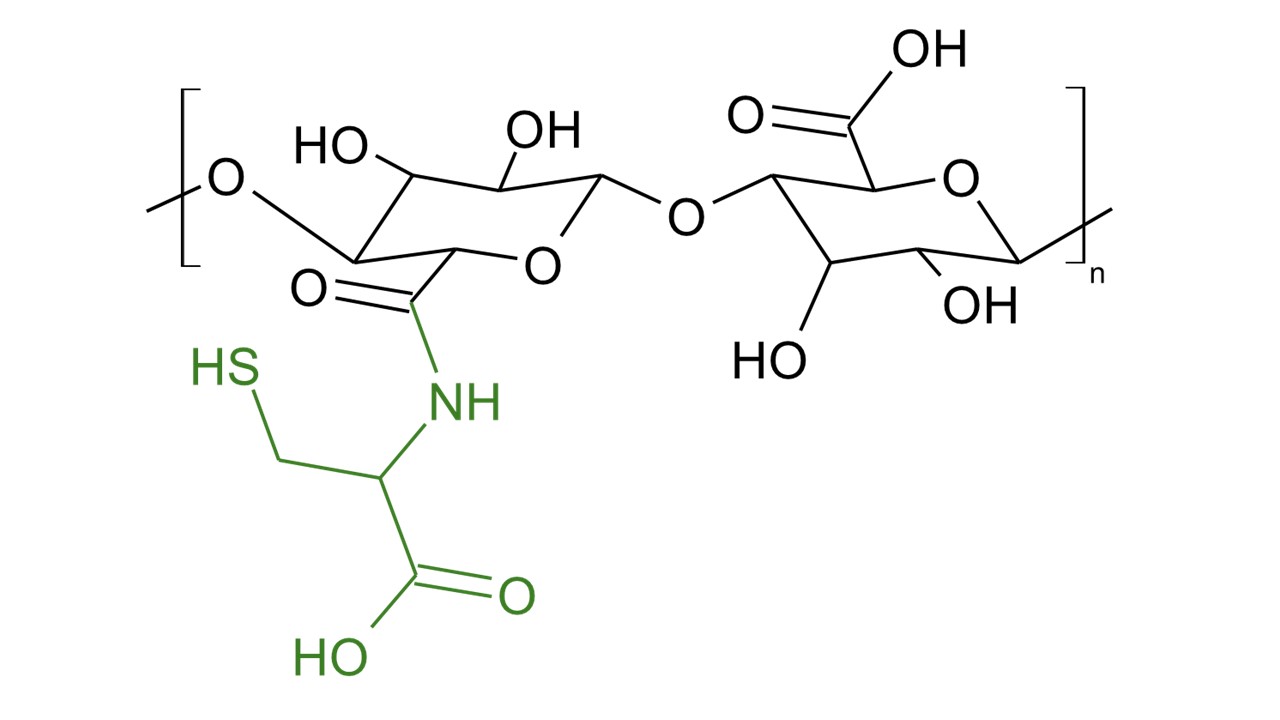


**Figure S3.** Chemical structure of alginate/L-cysteine conjugate.


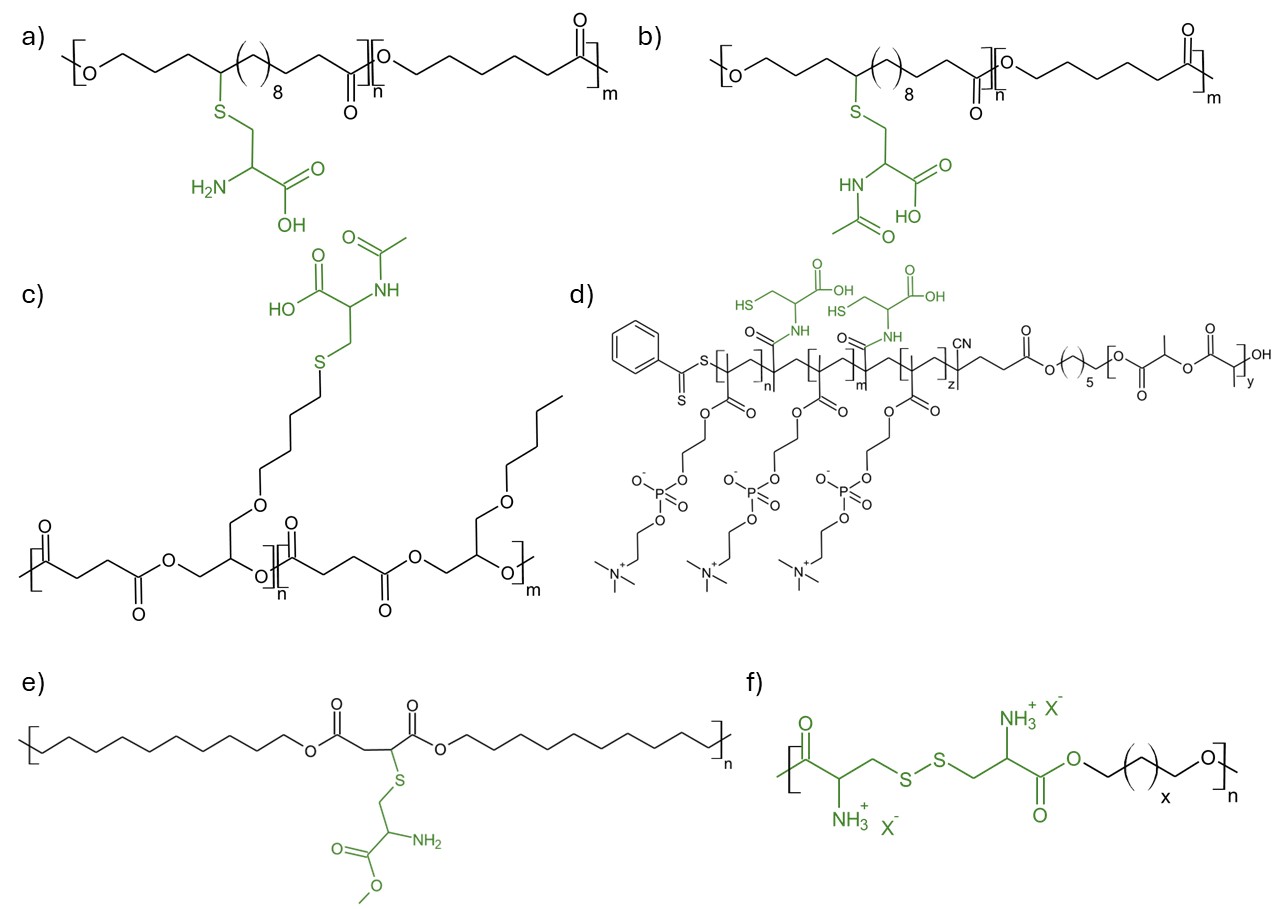


**Figure S4.** Chemical structure of polyester based conjugates: a) poly(globalide-co-ɛ-caprolactone)/L-cysteine, b) poly(globalide-co-ɛ-caprolactone)/N-acetyl-L-cysteine, c) polysuccinates/N-acetyl-L-cysteine, d) poly(L-lactide)-b-poly(2-(methacryloyloxy)ethyl phosphorylcholine)-st-(methacrylic acid NHS ester)/L-cysteine, e) di(10-undecenyl) maleate/L-cysteine methyl ester, f) polyester based on L-cystine monomer and diols.


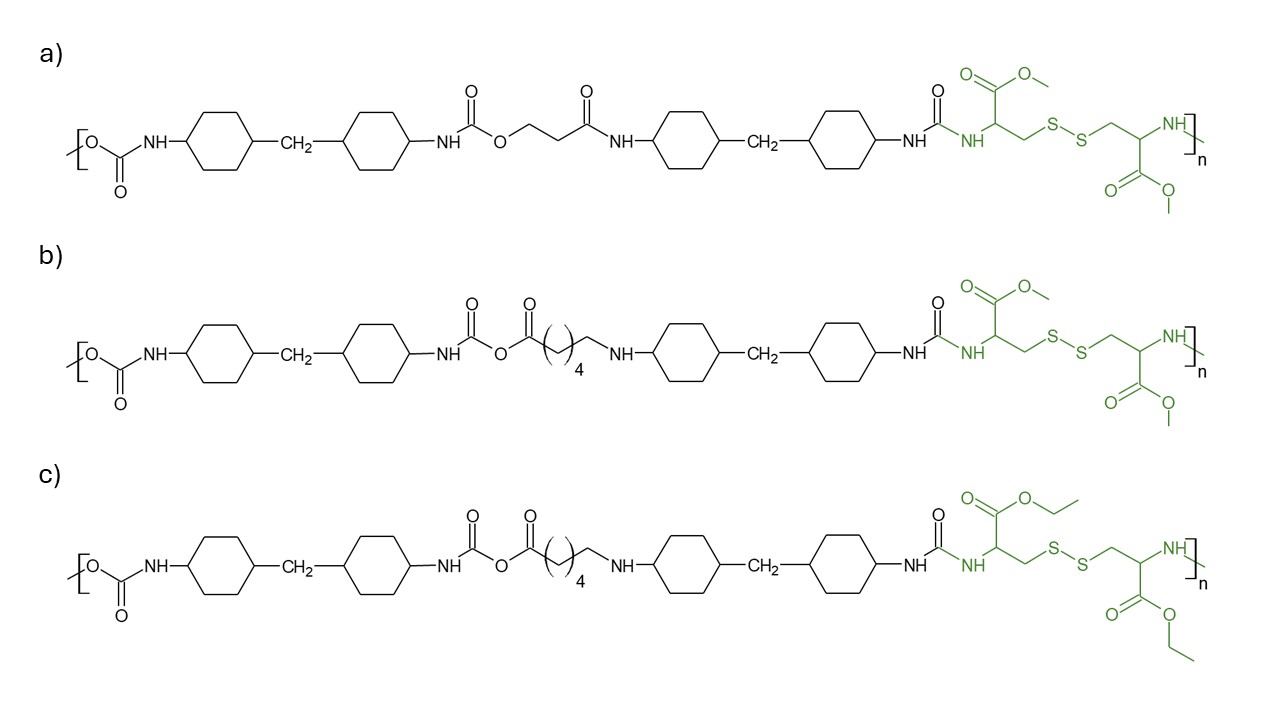


**Figure S5.** Chemical structure of polyurethane based conjugates: a) HMDI/PCL/L-cystine methyl ester, b) HMDI/PCL/L-cystine ethyl ester, c) HMDI/PEG/L-cystine methyl ester.


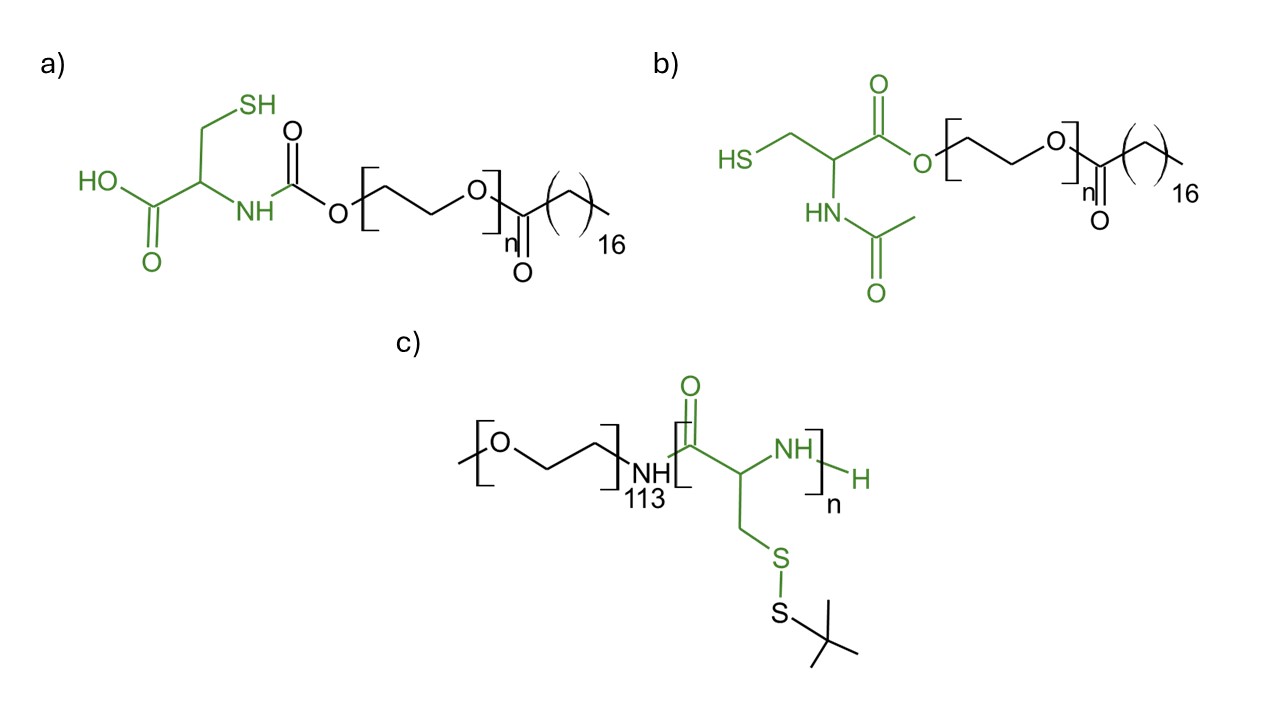


**Figure S6.** Chemical structure of poly(ethylene glycol) based conjugates: a) poly(ethylene glycol)/L‑cysteine, b) poly(ethylene glycol)/N-acetyl-L-cysteine, c) copolymer of poly(ethylene glycol) and polycysteine.


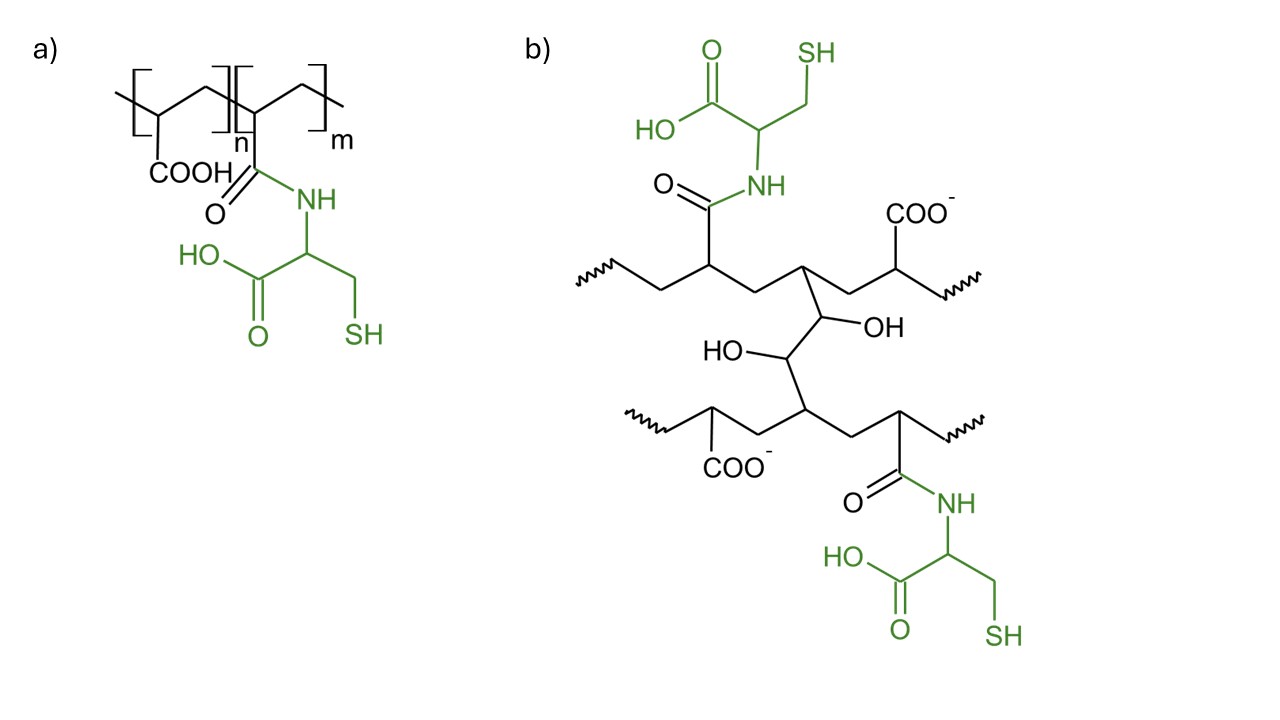


**Figure S7.** Chemical structure of a) poly(acrylic acid)/L-cysteine and b) polycarbophil/L-cysteine conjugates.


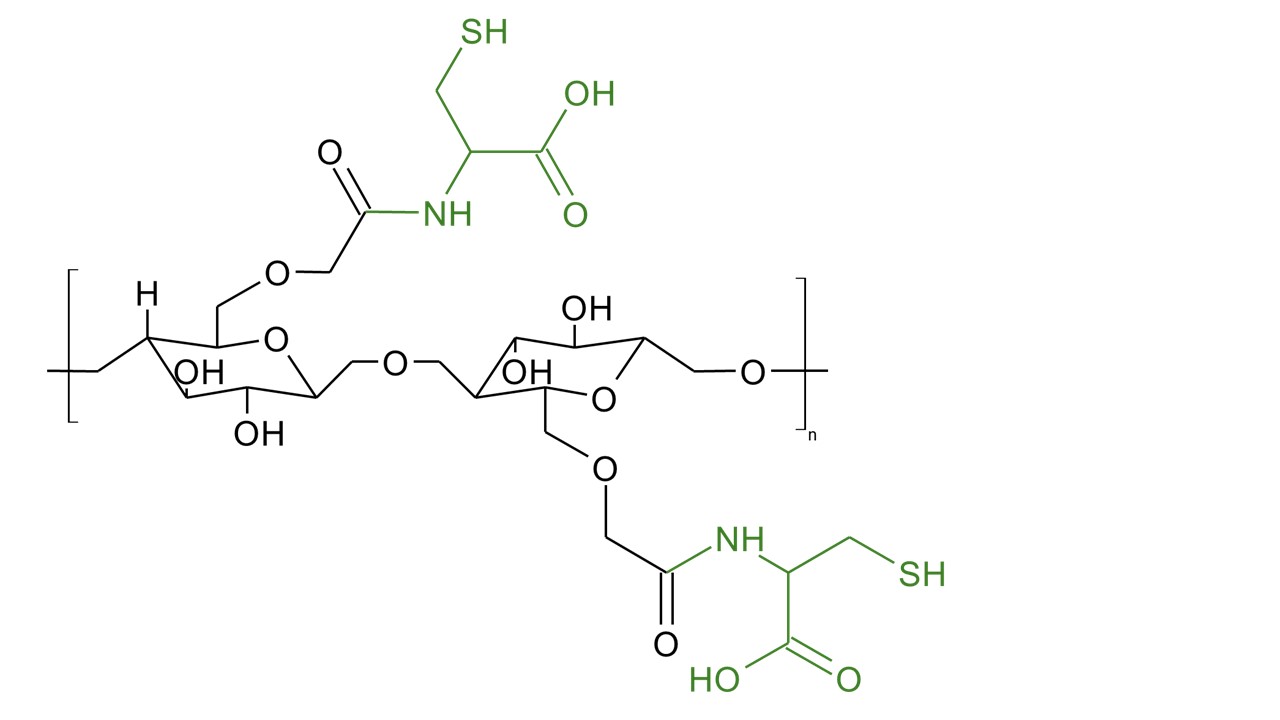


**Figure S8.** Chemical structure of carboxymethylcellulose/L-cysteine conjugate.
